# Editorial: “No Words for Feelings, Yet!” Exploring Alexithymia, Disorder of Affect Regulation, and the “Mind-Body” Connection

**DOI:** 10.3389/fpsyt.2020.593462

**Published:** 2020-09-18

**Authors:** Domenico De Berardis, Michele Fornaro, Laura Orsolini

**Affiliations:** ^1^ NHS, Department of Mental Health, Psychiatric Service for Diagnosis and Treatment, Hospital “G. Mazzini”, Teramo, Italy; ^2^ Section of Psychiatry, Department of Neuroscience, Reproductive Sciences, and Dentistry, Federico II University, Naples, Italy; ^3^ Section of Psychiatry, Department of Clinical Neurosciences/DIMSC, Polytechnic University of Ancona, Ancona, Italy; ^4^ Psychopharmacology, Drug Misuse and Novel Psychoactive Substances Research Unit, School of Life and Medical Sciences, University of Hertfordshire, Hatfield, United Kingdom

**Keywords:** alexithymia, emotional regulation, mind-body connection, affective dysregulation, psychosomatic and emotional difficulties

“*Unexpressed emotions will never die*.

*They are buried alive and will come forth later in uglier ways*.” (Sigmund Freud)

The term “alexithymia” was introduced by Peter Sifneos in 1973, to designate a cluster of cognitive and affective characteristics that were observed among patients with classical psychosomatic diseases (1). The alexithymia construct was further expanded over the years, resulting from many clinical investigations (2, 3). The construct is multifaceted and dimensional and includes several distinct characteristics: (a) difficulty in identifying and describing feelings, (b) difficulty in distinguishing feelings from the bodily sensations, (c) diminution of fantasy, and (d) concrete and poorly introspective thinking (4). The persons with alexithymia may show affective dysregulation, the inability to self soothe, and manage emotions because of a lack of awareness of emotions (5). Therefore, the adaptive informational meaning of emotions that is essential for emotion regulation often lacks in these individuals (6).

These cognitive features have been ascribed to a compromised capability to elevate emotions from a sensorimotor level of experience to a representational level, where they can be used as signaling reactions to internal or external events and moderated by psychological mechanisms (7). It has been demonstrated in several studies that often persons with alexithymia show significantly higher levels of psychological distress than persons without alexithymia and may develop “functional” somatic symptoms and complaints (8).

As a distinctive trait, the classical features of alexithymia are more evident during meaningful social interactions with high emotional valence (9). The affect-avoiding relational pattern behavior exhibited by the persons with alexithymia is habitually maladaptive. It may stimulate problems and conflicts in significant relationships, finally subsidizing the risk of the emergence of psychiatric symptoms such as depression and anxiety (10).

Moreover, alexithymia is a relatively stable personality trait, enhancing vulnerability to depressive symptoms, and is generally associated with a higher risk of death for several causes (suicide, accidents, injury, or violence) and is accompanied by an affective dysregulation (11, 12). The affective dysregulation may further impair emotion recognition, further increasing the risk of impulsive/addictive behaviors and the emergence of suicidal ideation and behaviors (13).

As an ideal introduction to the Research Topic, López-Muñoz and Pérez-Fernández provided a comprehensive and intriguing history of the alexithymia concept. They pointed out that the alexithymia construct may be understandable when observed from an epistemological perspective, and it mainly involves a rerun of the age-old conflict between basic and applied science, which in this case takes the form of a confrontation between the therapist’s clinical reflection and the critical judgment of experimental methodology.

Alexithymia is the quintessence of impairment of “mind-body connection,” and its recognition is crucial in everyday clinical practice to develop *ad hoc* interventions, considering that persons with alexithymia and concurrent psychiatric disorders are often treatment-resistant (not only to pharmacotherapy but also to psychotherapy) (14, 15). Concerning this point, several papers have explored the issue of treatment resistance and possible interventions. Pinna et al. performed a systematic review to evaluate the shreds of evidence on the role of alexithymia role in influencing the treatment outcome in several psychiatric disorders. They found that the existing data tend principally to correlate low baseline, and/or post-treatment levels of alexithymia and/or an improvement in levels of alexithymia throughout treatment, with a more satisfactory outcome of the treatment of the psychiatric disorders considered. Following this observation, Gramaglia et al. reviewed the treatment outcome in Anorexia Nervosa (AN) in terms of changes in alexithymia as assessed by its most commonly used self-report measure, the Toronto Alexithymia Scale (TAS). They found that a relevant percentage of persons with AN and comorbid alexithymia reached only partial recovery following treatment. Therefore, the identification of outcome predictors such as alexithymia is essential, as well as that of treatments specifically targeting such predictors.

Accordingly, one can argue that pre- and during-treatment interventions aimed to reduce alexithymia levels may be somewhat beneficial to achieve an excellent therapeutic response (16, 17). Aaron et al. assessed the relationships between alexithymia and interoceptive accuracy and sensibility in a sample of healthy young adults and the outcomes of a brief mindfulness-based body scan intervention. The results showed that higher alexithymia levels might be associated with either relatively high or low interoceptive accuracy. They also showed that the meditation mindfulness-based body scan intervention did not result in improved interoceptive accuracy or sensibility above and beyond that of a control group. Therefore, improvements in interoceptive accuracy, interoceptive sensibility, and emotional awareness may require longer or more interactive intervention approaches.

Interestingly, in a Perspective Article of this Research Topic, Shalev suggested that clinicians and neuropsychologists could help individuals who suffer from alexithymia by coaching them to use awareness-of-sensation practices. She argued that these techniques might promote relaxation or self-acceptance, also facilitating interoceptive improvement through an improved distinction between bodily perceptions and psychological interpretation. Besides, as demonstrated by Fournier et al., difficulties in interoceptive abilities (DIA) were predictive of the presence of irritable bowel syndrome (IBS), suggesting that when faced with a new stressful situation, patients in remission would have difficulty in perceiving their internal body changes and thus would have difficulty properly regulating their emotions. On the other hand, in the Crohn’s disease group, difficulties in describing feelings dimension of alexithymia were found a predictor of the presence of this disorder. In contrast, in the Ulcerative Colitis group, predictors seem to belong more to the physiological sphere rather than alexithymia. These results were encouraging and opened new perspectives for the healthcare of patients with gastrointestinal diseases, such as the use of Cognitive Behavioral Therapy. Duquette presented a very interesting case series describing the disturbances in emotional awareness in alexithymia, trying to integrate psychotherapeutic and neuroscience findings. Considering the current advances in neuroscientific theory and its application to psychotherapy, she argued that persons with alexithymia should be trained and enhanced in the ability to switch their attention between: their inward interoceptive sensations flexibly; exteroceptive sensory signals from the world, in the present moment (i.e., the context); and their own usual and deeply held prior beliefs about the causes or meanings of sensations.

Several contributions in this Research Topic also aimed to evaluate alexithymia in clinical and non-clinical samples, further contributing to its understanding.

Concerning non-clinical samples, Elkholi et al. evaluated the rates of alexithymia and its relationship with smartphone addiction in a cross-sectional study on a sample of 200 university students and found a strong association between both. These results were consistent with most of the previous literature that pointed out that persons with alexithymia tend to regulate their emotions through several forms of addictive behaviors. Kajanoja et al. examined the relationships of alexithymia and hair cortisol concentrations (HCC), a measure of long-term cortisol levels, and a biomarker for chronic stress in pregnant women. They found that among pregnant women, moderate to high alexithymia was associated with elevated HCC levels, and this association was driven by the subjective difficulty in identifying feelings (DIF) dimension of alexithymia, the only associated with HCC in the whole sample, after controlling for potential confounders. These results are in line with the so-called “stress-alexithymia hypothesis” (18, 19). Ma et al. explored the relationships between schizotypal traits, alexithymia, and sleep problems in 2,626 first-year students of a medical university. They found positive correlations between schizotypal traits, alexithymia, and sleep difficulties, although alexithymia was found to mediate the relationship between schizotypal traits and sleep difficulties partly. They also suggested that training the ability to express emotion may be capable of improving the sleep quality of persons with higher schizotypal traits.

Concerning clinical samples, Raffagnato et al. evaluated 134 adolescents with “nonsuicidal self-harming, or self-injury” (NSSI). They found that individuals who engaged in NSSI had more problems in the internalizing and externalizing domains than the control group, and internalizing disorders (and affective disorders in particular) was the factor that most strongly characterized the clinical group. Moreover, self-harming persons had more severe somatic disorders and alexithymia. These findings demonstrated that alexithymic traits are linked to the use of the body instead of words to express emotion in the case of psychological disease and may explain body NSSI when the emotional dysregulation and negative feelings are overwhelming. Bileviciute-Ljungar and Friberg investigated the connections between the alexithymia (using the TAS-20) and the level of emotional awareness, as well as depression and anxiety, with objective sleep parameters from polysomnography in patients with myalgic encephalomyelitis/chronic fatigue syndrome (ME/CFS). The results of this pilot study indicated a biological mechanism such as sleep fragmentation that may affect emotional awareness.

Moreover, they hypothesized that alexithymia and emotional awareness might have different associations with sleep and probably are regulated in different ways, though overlapping each other. On the other hand, Šago et al. assessed the difference in the prevalence of alexithymia in panic disorder (PD) and other anxiety disorders (OAD). They found that the prevalence rate of alexithymia in PD was 27%, and the difficulty identifying feelings (DIF) subscale scores of TAS-20 DIF was significantly higher in the PD group than in the OAD. Because of these results, they argued that higher rates of alexithymia in PD might reflect a person’s disposition to tighten their emotional experience to elude affect-based psychological sensations. These results are in line with those of previous studies on PD (20, 21).

As an ideal conclusion of the present Research Topic, the paper by Tonello et al. explored fascinatingly the “mind-body connection” and investigated the relationships between depression and objective measures of body fatness, autonomic indices, cardiorespiratory fitness and physical activity levels in 35 non-exercising women. They found that that cardiorespiratory fitness was the best predictor of depressive symptoms independently of body fatness, with maximum oxygen consumption (VO2max) and the sum of skinfolds moderately related to depression scores and VO2max the only independent predictor of depression in several stepwise multiple linear regression models.

In conclusion, all the papers in the present Research Topic provided clarification on the alexithymia construct and the “mind-body connection,” also trying to give practical and useful information on their evaluation and possible treatment options. We believe that alexithymia and its impact on “mind-body connection” still represent a challenge for all mental health and healthcare workers. Still, there is a silver lining as early detection of alexithymia has become more frequent in everyday “real-world” clinical practice (22). We hope that the research on these fundamental topics in mental and general health will proceed to explore this construct further and to find an appropriate and reliable therapeutic strategy.

## Author Contributions

The Editorial has been written by DB, MF, and LO.

## Conflict of Interest

The authors declare that the Editorial was written in the absence of any commercial or financial relationships that could be construed as a potential conflict of interest.
